# Neuroendoscopic lavage: a single-center retrospective cohort in the USA

**DOI:** 10.1007/s00381-025-06994-z

**Published:** 2025-11-13

**Authors:** Sunny Abdelmageed, Lucinda Chiu, Maria Dizon, Ulrich-Wilhelm Thomale, Jeffrey S. Raskin, Michael DeCuypere, Jonathan Scoville, Sandi Lam

**Affiliations:** 1 Division of Pediatric Neurosurgery, Ann and Robert H. Lurie Children’s Hospital of Chicago, 225 E Chicago Ave, Box 28, Chicago, IL USA; 2 Department of Neurological Surgery, Northwestern University Feinberg School of Medicine, Chicago, IL USA; 3 Department of Neurological Surgery, Rush University Medical Center, Chicago, IL USA; 4 Division of Neonatology, Ann and Robert H. Lurie Children’s Hospital of Chicago, Chicago, IL USA; 5 Division of Neonatology, Northwestern University Feinberg School of Medicine, Chicago, IL USA; 6 Pediatric Neurosurgery, Charité-Universitätsmedizin Berlin, Freie Universität Berlin, Humboldt-Universität Zu Berlin, Berlin Institute of Health, Berlin, Germany

**Keywords:** Intraventricular hemorrhage, Posthemorrhagic hydrocephalus, Prematurity, Ventriculosubgaleal shunt

## Abstract

**Purpose:**

Neuroendoscopic lavage (NEL) has been described for post-hemorrhagic hydrocephalus management in intraventricular hemorrhage (IVH) of prematurity in European cohorts. We describe an initial single-center series from the USA.

**Methods:**

A retrospective review was performed for premature infants with IVH that underwent NEL at our institution between 2020 and 2023. Patient characteristics, clinical variables, and radiological assessments were collected.

**Results:**

Eleven patients (five female) with IVH grade III/IV underwent 13 procedures. Mean gestational age (GA) was 25 weeks and 2 days. Mean birth weight (BW) was 0.83 kg (kg). Average age at NEL was 40.4 ± 21.5 days, and mean weight was 1.51 ± 0.3 kg. Mean frontal horn index decreased from 0.68 to 0.56 after NEL (*p* < 0.001). Cerebrospinal fluid infection was diagnosed in 18.2%, secondary hemorrhage in 18.2%, and seizures in 27.3% of patients. One patient died postoperatively with refractory coagulopathy. Conversion to ventriculoperitoneal shunt at six-month follow-up was 8/11 (72.7%), with 50% 1-year revision-free shunt survival. No patients required a multi-catheter system. Rates of comorbidities and shunt dependency showed very strong positive correlations: hyaline membrane disease rate (*R*^2^ = 0.950), necrotizing enterocolitis (*R*^2^ = 0.999), and persistent ductus arteriosus (*R*^2^ = 0.975). Prematurity and shunt dependency showed a moderate to strong negative correlation: GA (*R*^2^ = 0.527) and BW (*R*^2^ = 0.344).

**Conclusion:**

Extreme prematurity and comorbidities are associated with increased shunt dependency. However, NEL may decrease the development of complex multiloculated hydrocephalus and the need for multiple catheter systems. Larger, long-term studies are needed to define optimal timing and criteria for NEL and its benefits and impact on neurodevelopment in this fragile population.

**Supplementary Information:**

The online version contains supplementary material available at 10.1007/s00381-025-06994-z.

## Introduction

Intraventricular hemorrhage (IVH) is a serious complication of prematurity, impacting 20–40% of premature births weighing less than 1.5 kg worldwide [1]. The risk of severe IVH is inversely associated with infant maturity, as represented by gestational age (GA) and birth weight (BW), with mortality rates up to 40% [2].

Several factors contribute to the morbidity and mortality of severe IVH, including injury from the primary hemorrhage and secondary complications. Among patients with IVH, 25–50% of infants weighing less than 1500 g will develop post-hemorrhagic hydrocephalus (PHH) requiring neurosurgical intervention [3–5]. IVH may elevate concentrations of transforming growth factor-β (TGF-β) and other cytokines, potentially exacerbating cerebral damage and neuroinflammation [6, 7].

Neurosurgical treatments for IVH include lumbar punctures, ventricular aspiration, and temporizing procedures with hardware, including ventricular access devices (VAD) and ventriculosubgaleal shunts (VSGS) [8]. Despite multiple studies and guidelines, there is no treatment consensus [9]. Drainage, irrigation, and fibrinolytic therapy (DRIFT) was a randomized controlled trial that, despite early discontinuation due to hemorrhage-associated morbidity, did show decreased shunt dependency compared with standard treatment through 10-year follow-up [7, 8]. Neuroendoscopic lavage (NEL)—guided by the DRIFT framework—facilitates removal of early IVH blood products via controlled endoscopy, with lower rebleed rates, favorable outcomes in multiple European cohorts, and level III CNS guideline support [9–16].

Despite multiple European studies, there is a paucity of studies from North America. A recent study conducted in the USA reported an initial favorable experience with NEL [17]. Variations in neonatal populations and practices between the USA and Europe may reveal cohort differences impacting IVH severity and outcomes. We report our initial experience with NEL in a US cohort of patients with IVH of prematurity and PHH.

## Methods

### Patient selection

**Fig. 1:**
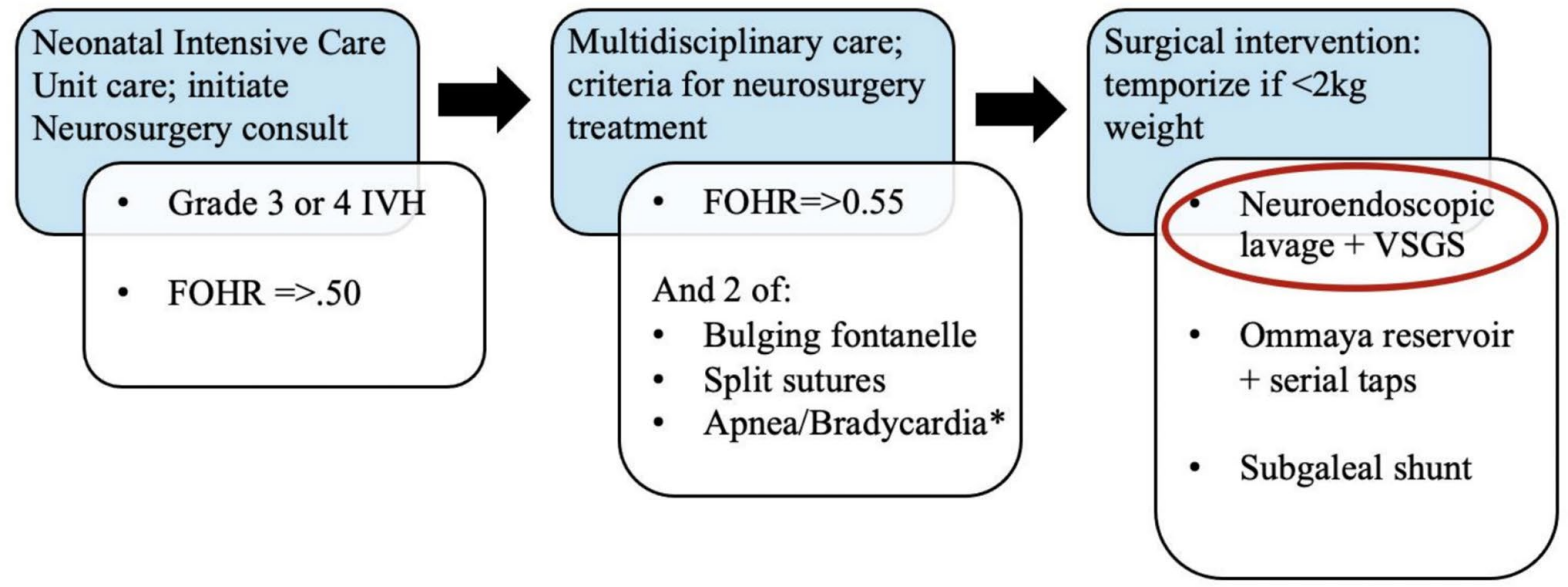
The multidisciplinary care pathway utilized at our institution for the management of patients with premature IVH. Indications for neurosurgery consultation, neurosurgical intervention, and types of neurosurgical intervention are listed. This pathway was adapted to our institution from the hydrocephalus clinical research network criteria published in Wellons et al. 2017, and clinical decision-making by our neonatal intensive care unit team. *Our VSGS systems included a Rickham Reservoir with the option for serial taps if needed

All very preterm neonates (< 32 weeks) with IVH of prematurity and PHH who underwent NEL at our institution from 2020 to 2023 with a minimum of eighteen months’ follow-up were included for retrospective chart review. Indications for neurosurgical evaluation were based on our modification of the Hydrocephalus Clinical Research Network (HCRN) clinical care pathway [18]. Bradycardia was defined as < 100 bpm and was determined to be clinically relevant as three or more prolonged episodes in a 24-h period that could not be explained by another medical condition as determined by our neonatal intensive care unit (NICU) clinical team. Each surgical intervention was based on a multidisciplinary neonatology/neurosurgery collaboration (Fig. 1). Patients with grade I/II IVH, hemodynamic instability, and/or uncorrectable coagulopathy were excluded from NEL. This study was approved by our Institutional Review Board (IRB #2023–6158).

### Endoscopes

**Fig. 2:**
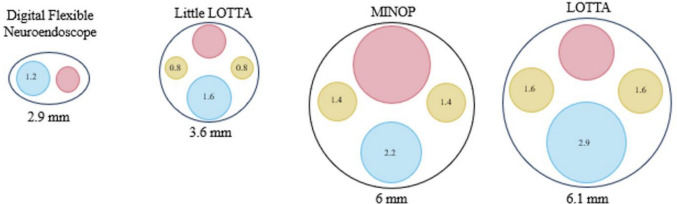
Representation of neuroendoscopes utilized for NEL in order of smallest to largest size (left to right). Each endoscope working channel is color-coded here. The working portal (blue, left/bottom) allows different instruments to be passed through, including a bipolar or grasping forceps. The optic channel for visualization is shown in pink (right/top), and the inflow/outflow portals for irrigation and CSF are shown in yellow (left/right). Of note, the flexible endoscope does not have an inflow/outflow portal. All scopes are from Karl Storz [Storz, Tuttlingen, Germany], except the Minop scope [Aesculap, Tuttlingen, Germany]

Flexible (Storz, Tuttlingen, Germany) and rigid endoscopes (Minop Rigid [Aesculap, Tuttlingen, Germany], Lotta and Little Lotta [Storz, Tuttlingen, Germany]) were used, as determined by surgeon preference and equipment availability (Fig. 2).

### NEL procedure

**Fig. 3:**
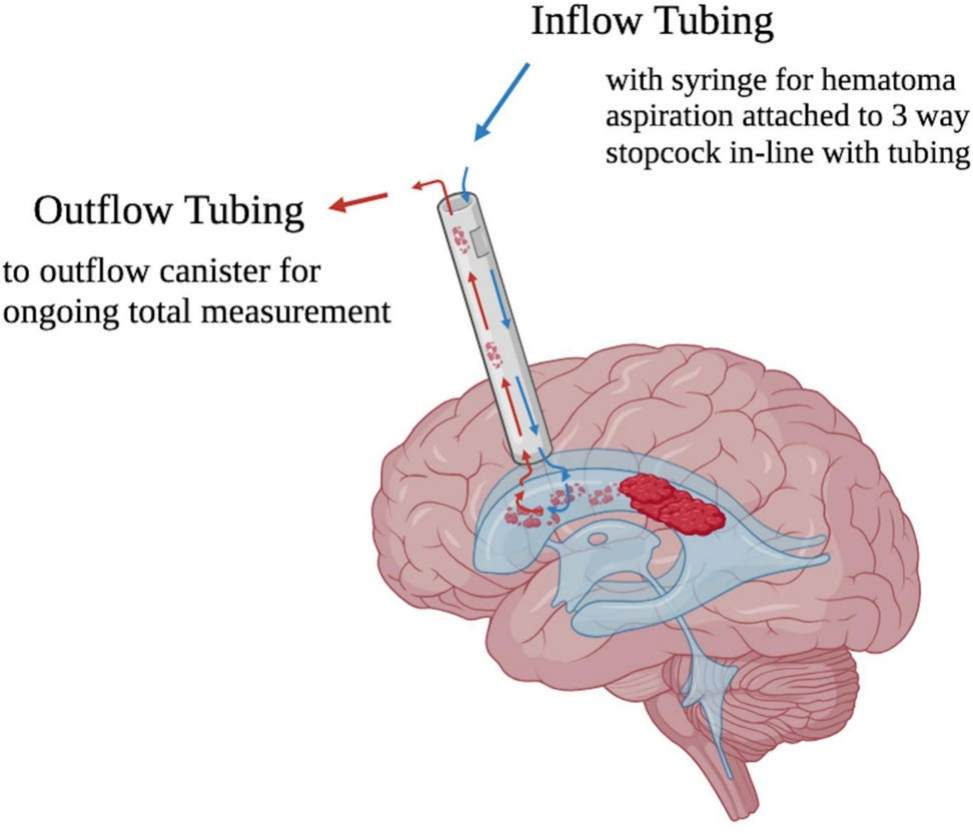
This figure demonstrates the neuroendoscopic lavage procedure (NEL). The brain is depicted from a lateral view. The neuroendoscope is depicted in gray, with two sets of arrows visible—blue (right) to represent the inflow of saline irrigation during the procedure and red (left) to represent the outflow of bloody CSF. Tubing for inflow and outflow, neuroendoscope camera, and syringe attached to a 3-way stopcock in-line with tubing are not shown here

Under general anesthesia, patients were placed supine with a shoulder bump to enable their head to be comfortably presented at 90° lateral, approach side facing up, on a DORO (Pro Med Instruments GmbH, Freiburg, Germany) headholder system. Thorough plastic draping kept the infant’s body warm and dry during procedural irrigation. Weight-based dosing of Ancef was given within 1 h of skin incision. The frontal incision was made approximately 2 cm off midline, at the coronal suture, and at the anterior fontanelle. The endoscope was inserted into the lateral ventricle with ultrasound guidance. Laterality was determined based on the amount of clot burden provided that the anatomical configuration allowed for safe access. Once bloody or hemosiderin-laden cerebrospinal fluid (CSF) was encountered, warm normal saline irrigation was initiated to assist with visualization of expected anatomic landmarks for orientation. Blood products were removed via the endoscope working portal by applying gentle manual syringe suction proximally. A septostomy, if needed, allowed access to the contralateral ventricle to repeat this process. Septostomy was accomplished with a brief bipolar electrocautery to create the stoma in the septum after careful selection and confirmation of an avascular area 1–2 cm superior-posterior to the venous angle. Warm irrigation (37–38 °C) was continued until it ran clear. Following endoscope removal, the intraventricular catheter of the VSGS system was placed through the same dural opening, and the VSGS hardware configuration in our practice includes a Rickham tapping reservoir to function as a VAD as well. In the procedure for placing this VSGS system, the tapping reservoir and patent opening distal to the reservoir to allow for CSF egress were placed in the subgaleal space (Fig. 3).

### Postoperative care

Multidisciplinary management is essential for postoperative care following NEL. Screening video electroencephalography (vEEG) was part of our clinical pathway to avoid over- or under-treatment with anti-epileptics. All patients were placed on vEEG on postoperative day 0 (POD0), and removal of EEG leads occurred either after 48 h of no electrographic epileptic activity or after adequate treatment and resolution of electrographic abnormalities with additional antiepileptics, as needed. Head ultrasound (HUS) was performed at least weekly with de-escalation over time if the patient’s neurologic exam remained stable. If clinical or radiographic signs of hydrocephalus recurred, additional CSF diversion procedures were considered, including repeat NEL, VSGS revision, endoscopic third ventriculostomy (ETV) with or without lysis of adhesions, and/or placement of a shunt. Continued bedside temporization for infants < 2 kg most commonly involved VSGS reservoir taps since a tapping reservoir was included in the VSGS configuration and could be accessed for serial CSF tapping even after eventual obliteration of the subgaleal pocket.

### Patient factors

Patient risk factors such as GA, BW, pre-operative modified Papile grade, and comorbid conditions were reviewed. Comorbid conditions included hyaline membrane disease (HMD), persistent ductus arteriosus (PDA), necrotizing enterocolitis (NEC), meningitis (suspected or proven), and seizures.

### Perioperative events

Perioperative events included CSF infection (defined by positive CSF cultures), secondary hemorrhage (defined by new/increased blood volume on imaging within 72 h following surgery), and postoperative subclinical electrographic seizures (defined by detection on routine screening vEEG by the neonatal neurology team).

### Radiographic assessment and analysis

IVH severity was described using the modified Papile classification [19]. Grading was performed by pediatric neuroradiologists, or if unavailable, the study team. The frontal occipital horn ratio (FOHR) and ventricular index (VI) were measured from the most recent preoperative HUS (within 72 h) prior to NEL and the first postoperative HUS. FOHR is defined as the sum of the frontal horn width and the occipital horn width divided by two times the biparietal diameter measured on axial images. VI is the distance between the lateral wall of the anterior horn and the falx. While our multidisciplinary clinical workflow and communication were based mainly on FOHR measurements, VI is also discussed here in this manuscript for comparison with published literature.

### Ventriculoperitoneal shunt (VPS)

The decision to convert to VPS placement after NEL + VSGS was based on team consensus. Criteria included patient weight > 2 kg, with FOHR > 0.55 increasing over time, and progressive failure of temporizing measures, determined by subgaleal pocket shrinking and/or progressing to requiring serial taps, along with two clinical symptoms of increased ICP as presented in Fig. 1. Shunt revisions were recorded for patients who received a VPS.

Patients that were > 2 kg but had a hostile distal site may have undergone a repeat temporizing measure. The abdomen is considered unusable in the setting of active NEC, diverting ostomy with anticipated repair, unrepaired omphalocele or bladder exstrophy, or any abdominal issue that may inhibit CSF absorption. VA sites can be viable in a premature infant. The patients in our series who underwent a repeat temporizing procedure at > 2 kg had specific reasons that inhibited VP and VA shunt sites. For instance, one patient had prolonged central line access for chronic TPN due to liver failure, and a VA shunt was not recommended at that time by the pediatric surgery service.

### Statistical analysis

Delta was calculated by subtracting the preoperative measurement of VI and FOHR from the postoperative values, respectively. A paired, one-tailed Student’s *t*-test was used to compare pre- and postoperative FOHR and VI. *P* ≤ 0.05 was considered significant. Linear regression was performed with mean prematurity and comorbidities as independent variables and mean shunt dependency rate as a dependent variable. *R*^2^ values were interpreted using the following determinations: very strong (0.7–1.0), strong (0.4–0.69), moderate (0.3–0.39), and weak (0.2–0.29). Statistical analysis was completed using RStudio (RStudio Team, 2023). Studies were included in the linear regression if mean values for shunt dependency were independently reported for an NEL-only group.

## Results

### Patient characteristics

Eleven infants (five female), with BW < 1.5 kg, underwent NEL + VSGS between June 2020 and September 2023. In our cohort, 2/11 patients (18.2%) had grade III IVH, and 9/11 (81.2%) had grade IV IVH. The mean FOHR was 0.51 ± 0.08 at the time of consultation. All patients showed clinical and imaging findings of worsening ventriculomegaly meeting criteria for CSF diversion via a temporizing measure (Fig. 1). Patient demographics are described in Table 1.

**Table 1:** Demographics and surgical characteristics

Case no	GA, sex	BW/Weight at Sx (g)	Calendar age/PMA	Endoscope	Temporizing device	Operative time (mins)	LOS	Follow-up (days)
1	24w4d, M	0.89/1.68	47/31w2d	Flexible Storz	VSGS	92	258	1104
2	24w2d, F	0.75/1.51	40/30w	Flexible Storz	VSGS	113	289	1223
3	23w0d, F	0.61/0.81	28/27w	Flexible Storz	VSGS	43	140	873
4	24w0d, F	0.60/1.59	46/30w4d	Minop Rigid	VSGS	61	68	737
5	25w1d, M	0.72/1.71	53/32w5d	Lotta Rigid	VSGS	65	54	54
6	25w3d, M	0.83/1.65	57/33w5d	Minop Rigid	VSGS	71	217	522
7	25w5d, M	0.65/1.54	41/31w4d	Minop Rigid	VSGS	49	146	475
8	27w5d, M	1.18/1.24	15/29w6d	Little Lotta Rigid	VSGS	105	34	364
9	27w2d, F	1.07/1.26	18/29w6d	Lotta Rigid	VSGS	62	166	281
10	27w0d, F	1.10/1.04	14/29w	Little Lotta Rigid	VSGS	41	202	215
11	24w0d, M	0.74/2.58	84/36w1d	Flexible Storz	VSGS	41	221	184

*GA*, gestational age; *M*, male; *F*, female; *BW*, birth weight; *Sx*, surgery; *IVH*, intraventricular hemorrhage; *DOL*, day of life; *kg*, kilogram; *w*, weeks; *d*, days; *VSGS*, ventriculosubgaleal shunt; *LOS*, length of stay

The cohort self-identified as White (5/11, 45.5%), Black (2/11, 18.2%), Asian (1/11, 9.1%), and other (3/11, 27.3%). Hispanic/Latino was the reported ethnicity in 3/11 patients’ families (27.3%). Mean gestational age at birth was 25 weeks and 2 days ± 1.5 weeks, median 25 weeks and 1 day. Mean BW was 0.83 kg (range 0.6–1.1 kg); median BW was 0.75 kg. Comorbidities in this cohort included HMD in all cases, PDA in 10/11 (90.9%), NEC in 8/11 (72.3%), and meningitis in 2/11 (18.2%). Seizures were diagnosed by vEEG in 5/11 (45.5%) of the patients, with two patients’ seizures occurring only in the preoperative period. Prior to neurosurgical intervention, seven patients had coagulopathies and cytopenias necessitating blood product transfusions, and six patients required vasopressors to maintain normotension.

The average length of stay in the neonatal intensive care unit (NICU) was 158 ± 83 days, median 166 days. The mean in-hospital ventilation duration was 70.3 days (range 2–279 days); median 54 days. At discharge, 5/10 were on room air, 3/10 required nasal cannula with supplemental oxygen, and 2/10 were ventilator-dependent with a tracheostomy. Nutritional status at discharge included 1/10 taking oral feeds, 4/10 requiring a nasogastric tube, and 5/10 with a gastrostomy tube. The mean total follow-up was 29.5 months (range 1.8–52.7 months); median 28.5 months.

### Perioperative variables

Mean calendar age at the time of NEL was 40.4 ± 21.5 days, median 41 days (range 15–85 days); PMA mean 30w6d, median 30w4d (range 29–36w2d); mean weight was 1.51 ± 0.3 kg, median 1.54 kg (range 810–2580 g). One patient received an NEL at 2.6 kg due to prior bowel perforation and expected future abdominal surgeries, despite meeting VPS criteria. Mean operative time was 67.5 ± 26 min, median 62 min (range 41–113). Mean estimated blood loss was 1.5 ± 0.6 ml (median 2 ml) (Table 1). The Storz flexible endoscope was used in four patients, the MINOP Rigid endoscope in three patients, the Lotta Rigid in two patients, and the Little Lotta Rigid in two patients. Two patients received blood product transfusions, and one patient required vasopressors perioperatively.

### Perioperative events

Outcomes and perioperative events are shown in Table 2.

**Table 2:** Outcomes

Case #	FOHR ∆	VI (mm > 97th) ∆	POD at HUS	Operative time (mins)	Perioperative events	Clot burden remaining	Permanent CSF diversion
1	− 0.10	− 8.30	7	92	Preop seizure	Yes	Yes
2	− 0.26	− 9.10	10	113	Preop seizure, CSF infection	No	Yes
3	− 0.14	− 10.05	5	43	Local skin infection	No	No
4	− 0.06	− 4.85	3	61	Wound dehiscence	No	Yes
5	− 0.05	0.80	1	65	Hemorrhage, death	Yes	No
6	− 0.24	− 12.10	7	71	None	Yes	Yes
7	− 0.08	− 7.00	1	49	Preop seizure, vEEG seizure POD 1	Yes	Yes
8	− 0.11	− 4.25	1	105	CSF infection	Yes	Yes
9	− 0.11	− 3.00	4	62	vEEG seizure POD 3	Yes	Yes
10	− 0.06	2.60	7	41	None	Yes	Yes
11	− 0.08	− 6.30	3	41	vEEG seizure POD2, hemorrhage	No	No

**∆** represents the change in preoperative and postoperative FOHR or VI; *#*, number; *FOHR*, frontal-occipital horn ratio; *VI*, ventricular index; *LOS*, length of stay; *CSF*, cerebrospinal fluid; *vEEG*, video EEG; *POD*, post-op day

There was one mortality in a patient with grade IV IVH, multiple comorbidities, and redirection of goals of care. This patient was excluded from perioperative adverse event frequency calculations. A CSF infection in the postoperative period was diagnosed in two patients—one with *Enterobacter cloacae*, and another with methicillin-sensitive *Staphylococcus aureus*. Two patients had secondary IVH within two days postoperatively. Four (36.4%) patients required repeat temporizing measures (all with redo VSGS; two with and two without NEL) before definitive CSF diversion, as they had hostile distal CSF shunting sites or low body weight. Three (27.3%) patients had vEEG evidence of a seizure within 72 h postoperatively.

### Radiographic measurements

Cranial imaging in the form of HUS was obtained postoperatively (median = four days, mean = 4.45 days, range 1–10 days). The mean FOHR decreased from 0.68 prior to NEL to 0.56 after NEL (∆ − 0.12, median 0.66 to 0.53 − 0.13 *p* < 0.001, Table 2). The VI > 97th percentile decreased from 11.8 mm prior to NEL to 6.2 mm after NEL (∆ − 5.6, *p* < 0.001); median 9.6 to 6.2, 6.3 mm. Following NEL, all IVH grades were stable or improved: one patient had grade 0, three patients had grade I, three had grade II, one had grade III, and four had grade IV IVH.

### Permanent CSF diversion

Patients had an average follow-up of 30 months, median 28.5 (1.8–52.7) months. Eight patients thus far have required permanent CSF diversion. Seven patients had a VPS placed, and one had a ventriculoatrial shunt placed given a hostile abdomen. Mean time between NEL and shunt insertion was 149 days, median 81 days. Mean calendar age at shunt insertion was 6.1 ± 5.6 months, median 3.7 months, and the mean weight was 3.87 ± 1.8 kg, median 3 kg (range 2.6–6.6 kg). The mean number of revisions was 1.6. Four patients required at least one revision at 23, 28, 119, and 120 days. The remaining four patients have not required shunt revisions (mean 33 ± 10.4 months, median 31.5 (23.5–45.5) months). Revision-free shunt survival at 1 year was 50%. The Kaplan–Meier curve for revision-free shunt survival is shown in Supplementary Fig. 1. Four of eleven patients developed multiloculated hydrocephalus. Neuroendoscopic fenestration enabled all cases to be successfully managed by a single ventricular catheter. Neuroendoscopic fenestration was completed concurrently during another indicated procedure, either repeat NEL, ETV, or shunt placement at (5.6, 3.5, 2.9, and 7.7) months of age, (4.1, 2, 1, and 6.3) months after NEL. Permanent shunt placement occurred (12.6, 2.1, and 1) months after neuroendoscopic fenestration, with one occurring on the same day.

Compared with most European cohorts, our patients demonstrated higher shunt dependency rates (Table 3).

**Table 3:** Comparison with published cohorts

	Lurie	Schulz (2014)	D’Arcangues (2018)	Tirado–Caballero (2020)	Frassanito (2021)	Honeyman (2022)	Flanders (2025)
*N*	11	19	56	46	24	26	12^^^
Median GA at birth	25w1d (25w2d)	27w6d	26w6d	30w^*^	28w5d^*^	29w4d	25w0d (25w)
Median birth weight (kg)	0.75 (0.83)	1.04	1.11	1.67^*^	1.31^*^	1.41	0.75^^^ (0.78)
IVH grade							
II	0 (0.0%)	2 (10.5%)	5 (8.9%)	0 (0.0%)	–	1 (3.8%)	0
III	2 (18.2%)	13 (68.4%)	23 (41.1%)	28 (60.1%)	–	8 (3.1%)	–
IV	9 (81.2%)	3 (15.8%)	28 (50.0%)	18 (39.1%)	–	17 (65.4%)	–
Comorbid conditions							
HMD	11 (100%)	–	–	22 (47.8%)	–	18 (69.2%)	–
NEC	8 (72.3%)	–	–	5 (10.9%)	–	11 (42.3%)	–
PDA	10 (90.9%)	–	–	11 (23.9%)	–	17 (65.3%)	–
Median PMA at NEL	30.56 (30.86)	31.71w	30w4d	–	32.7w^*^	39.6w	29.82^^^ (29.82)
Median weight at NEL (g)	1.54 (1.51)	1.45	1.52	–	–	–	1.3^^^ (1.3)
Perioperative events							
CSF infection	2 (18.2%)	2 (10.5%)	2 (3.6%)	10 (21.7%)	1 (4.2%)	2 (7.7%)	1 (8.3%)^†^
Re-bleed	2 (18.2%)	–	5 (8.9%)	3 (6.52%)	–	1 (3.8%)	1 (8.3%)^†^
Death	1 (9.1%)	–	3 (5.4%)	3 (6.52%)	1 (4.2%)	0 (0.0%)	1 (8.3%)^†^
Permanent CSF diversion	72.7%	58.0%	56.6%	58.7%	87%	5.4%	66.7%^†^
Median time to shunt (w)	11.6 (10.4)	6.0	–	–	4.6^*^	10.14	9^^^ (10)
1-year shunt survival	50%	*	63.6%	50%	–	64.7%	–
Median # of procedures	2	2	2	–	–	–	–

Mean values reported in parenthesis when applicable. *, Manuscript only reported mean values; *–*, data was not available; *^*, Flanders et al. Only reported demographic information broken down by NEL vs non-NEL for 11 patients, therefore all values reported are as reported for 11 patients in table one and with a denominator of 11; *†*, these values have a denominator of 12 as reported in Table 3 of Flanders et al. *#*, number.

**Fig. 4:**
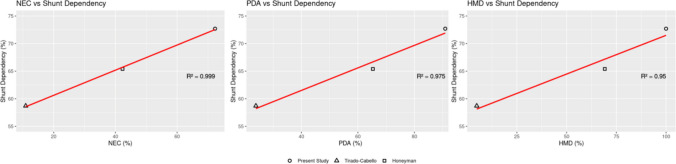
Graphs depicting linear regression performed for medical comorbidities (0–100%, *x*-axis) affecting shunt dependency rate (0–100%, *y*-axis) for all applicable cohorts. The linear regression line is in red, and the *R*^2^ value is depicted for each graph. As seen in the legend at the bottom, markers depict the study cohort: circle for the present study, triangle for Tirado–Caballero, and square for Honeyman. These depict very strong positive correlations between necrotizing enterocolitis rate (NEC) and shunt dependency (*R*^2^ = 0.999, left), patent ductus arteriosus rate (PDA) and shunt dependency (*R*^2^ = 0.975, middle), and hyaline membrane disease (HMD) and shunt dependency (*R*.^2^ = 0.950, right)

Linear regression between comorbidities and shunt dependency was performed using patient-level values reported in Tirado–Caballero et al. and Honeyman et al. [13, 14]. There was a very strong positive correlation between HMD rate (*R*^2^ = 0.950), NEC (*R*^2^ = 0.999), and PDA (*R*^2^ = 0.975), and shunt dependency (Fig. 4).

**Fig. 5:**
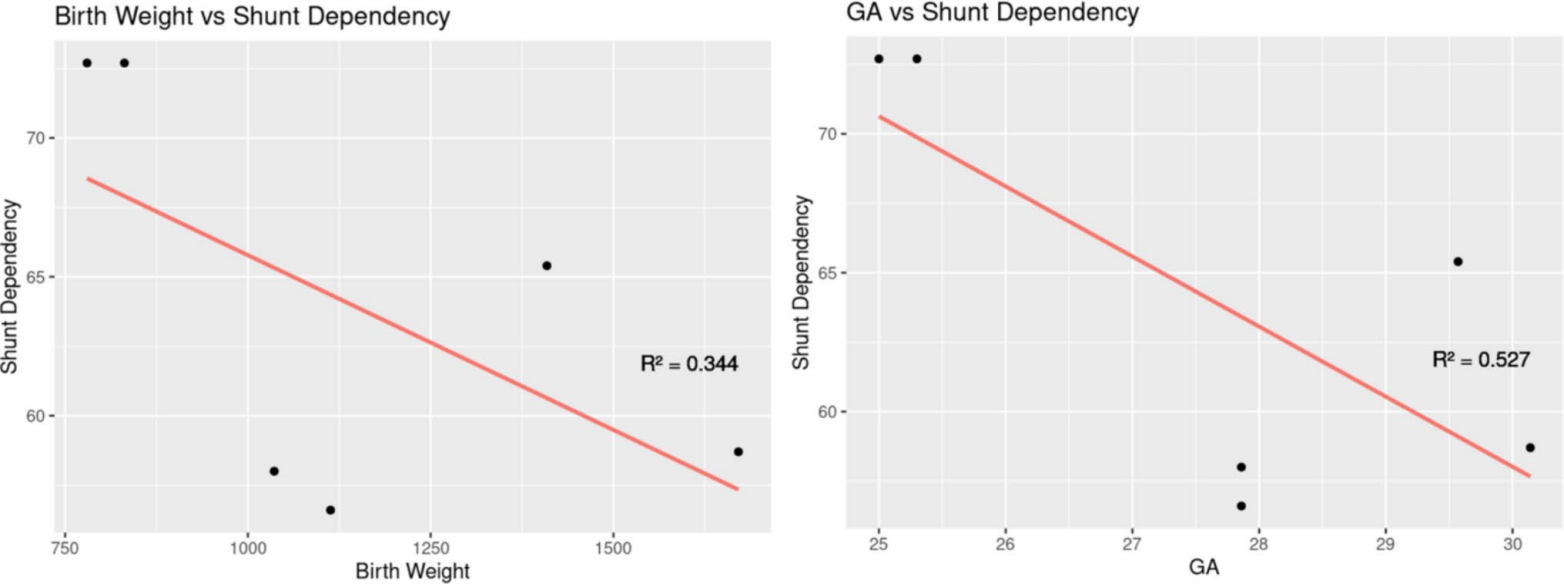
Graphs depicting linear regression performed for prematurity (*x*-axis) affecting shunt dependency rate (0–100%, *y*-axis) for all applicable cohorts. The linear regression line is in red, and the *R*^2^ value is depicted for each graph. The right graph depicts a moderate negative correlation between birth weight and shunt dependency (*R*^2^ = 0.380), while the left graph depicts a strong negative correlation between gestational age (GA) and shunt dependency (*R*.^2^ = 0.527)

Linear regression between prematurity and shunt dependency was performed using data from five published cohorts [10, 12–14, 17]. There was a strong negative correlation between gestational age and shunt dependency, GA (*R*^2^ = 0.527), and a moderate negative correlation between BW and shunt dependency (*R*^2^ = 0.344, Fig. 5).

## Discussion

This retrospective cohort study describes a single-center US experience with NEL. This US cohort is similar in average GA (25 weeks and 2 days) and BW (0.87 kg) to one published in another US institution (average GA 25 weeks, BW 0.78 kg) [17]. Prior studies have shown that the incidence of IVH is significantly higher in North America compared to European cohorts (*p*-value < 0.001), and that the incidence proportion of severe IVH for GA 25–27 weeks is higher in North America as well (North America: 0.22, Europe: 0.16) [20]. When compared with European cohorts, our patients are more premature with greater medical comorbidities. These differences may reflect variations in neonatal populations and practices between the USA and Europe [21]. Comorbid conditions in our cohort included higher rates of HMD, bronchopulmonary dysplasia, PDA, and NEC, which correspond with 55% of our patients being discharged on respiratory support and 91% with nutritional support via tube feeds. Our cohort also had a longer average mechanical ventilation duration (70.3 days) versus four days in the D’Arcangues et al. cohort [12].

Complication rates in this study are similar to previous NEL cohorts. Secondary hemorrhage was present in two patients (18.2%), which is higher than European cohorts (3.8–8.9%). This should be interpreted with caution given the small sample size, particularly as one case occurred in a patient with refractory coagulopathy and acute anemia prior to NEL. This rate remains lower than in DRIFT (35%), which carried a high risk of secondary hemorrhage due to the use of fibrinolytics [22]. These findings suggest that, as expected, NEL offers a more favorable safety profile in respect to secondary hemorrhage, though risks should be carefully weighed, particularly in patients with pre-existing coagulopathies. Screening vEEG detected postoperative seizures in 27.3% of our patients. This modality gives perspective on the incidence of perioperative seizures in these patients, data that has not been previously quantified or explored in published cohorts. It also allows for early seizure detection and timely treatment, which is critical, as the number of days with EEG-confirmed seizures has been identified as a potentially modifiable risk factor for developing postnatal epilepsy [23]. Our observed CSF infection rate was lower than the Spanish cohort and higher than the published UK and German cohorts [10, 12–14].

In our study, one patient died postoperatively. This mortality rate is consistent with previously published cohorts, reflecting the medical complexity and fragility of these patients, and underscoring the need for multidisciplinary care and ongoing research. A prospective study of the impact of IVH on mortality in very preterm (26–32 weeks GA) and low BW infants (< 1.5 kg) found that patients with grade III/IV IVH were 14 times more likely to die before NICU discharge compared with grade I/II IVH; surgical interventions for IVH were not reported [24]. In the North American HCRN Shunting Outcomes in Posthemorrhagic Hydrocephalus (SOPHH) study, 12.7% (13 of the premature infants < 1.5 kg BW) who underwent a VAD/VSGS died within six months postoperatively [18]. A 15-year retrospective study from our institution prior to adoption of NEL similarly found a 10% mortality (*n* = 3) after temporizing measures (VSGS/VAD) for grade III/IV IVH [25]. Among both the German and Spanish NEL series (*n* > 45), they each reported three deaths (6.6%) [12, 13]. Similarly, Flanders et al. reported one death (8.3%) among their US NEL patients [26]. Our mortality case had family wishes for the redirection of goals of care after encountering refractory coagulopathy, acute anemia, large-volume transfusions, and cardiopulmonary arrest.

Our rate of permanent CSF diversion was 72.7%, similar to published cohorts of 56–87.9% [12–15, 17, 27]. This rate is lower than shunt dependency rates reported in our prior retrospective study (77.8%) for grade III/IV IVH and other US studies after VAD/VSGS alone (90–95%) [3, 25, 28]. Flanders et al. whose US cohort had similar BW and GA to this cohort, also reported a similar permanent CSF diversion rate of 67% [17]. Severe prematurity is a significant risk factor for PHH and subsequent need for VP shunt insertion [28, 29]. Pooling data from the studies above, increased prematurity and medical comorbidities were associated with greater shunt dependency. The pathogenesis of IVH is complex, with multiple proposed mechanisms and risk predictors. Fluctuations in cerebral blood flow and impaired cerebral autoregulation, along with inflammatory cytokines, have all been implicated. Comorbidities of prematurity such as NEC, PDA, and HMD have previously been linked to these mechanisms and an associated increased risk of IVH, morbidity, and mortality [30, 31]. Consistent with these findings, in this study higher rates of comorbidities were associated with increased shunt dependency, likely explained by higher IVH severity and reduced compensatory mechanisms. A learning curve has also been described. Tirado–Caballero et al. observed a 72.72% rate of permanent shunting in their first 11 patients; in subsequent epochs in their experience, this rate dropped to < 54% [13]. A 2020 Italian retrospective study of 63 infants reported an 87.9% rate of permanent CSF diversion when comparing VSGS and VSGS ± NEL for PHH. Shunt dependency may also be influenced by the extent of IVH. Our series only included grade III/IV IVH patients, with 81.2% having grade IV. Grade IV IVH has a 52% increased risk of shunt dependency compared to grade II IVH, regardless of intervention [32]. European studies include patients with Grade II IVH; this variability precludes direct comparison.

Surgical timing in preterm infants with IVH involves a multidisciplinary risk–benefit assessment. While our time-to-intervention from birth to NEL (5.7 weeks) was longer than most European studies (3.95 weeks), it was more similar to the other US cohort (4.82 weeks). Previous studies have demonstrated improved outcomes and reduced shunt rates with earlier intervention [32–35]. Others have suggested ventriculomegaly indices thresholds of FOHR ≥ 0.66, VI ≥ 8.4 mm above the 97th percentile prior to CSF diversion [33]. We noted an increased time from NEL to shunt insertion of 10.4 weeks for those with NEL versus six weeks without NEL in our historical cohort [1, 10, 25]. The differences in GA at birth and comorbidity burden may also influence the timing of shunt insertion. The distal shunt site can be challenging in these patients: two had bowel resections with inhospitable abdomens and four had long-term venous catheters terminating in the right atrium. Ventriculopleural shunts are typically not considered in this age range and are further contraindicated by our patients’ respiratory complications of prematurity.

Another reported benefit of NEL is the reduction of complex hydrocephalus [12, 15]. Previously, conventional treatment cohorts have reported a median of 3.5 shunt revisions [10, 12]. Our study had a median of two neurosurgical procedures. While we recognize that secondary shunt failures are multifactorial and not always preventable, our NEL series has had the successful avoidance of multi-catheter multiloculated hydrocephalus. Four patients in our series underwent additional neuroendoscopic fenestration at the time of shunt revision, which has been durable thus far, with none requiring multiple catheter systems. In Etus et al. they reported an 8.6% multiloculated hydrocephalus rate in the NEL + EVD group compared with 40.9% in the VSGS-only group [27]. Similarly, the 1-year revision-free shunt survival in this small US cohort of 50% is similar to European cohorts, and it compares to historically reported rates of 44% in European and US cohorts [13–15, 32]. Neurodevelopmental outcomes will be discerned at longer-term follow-up. The Treatment of Post-Hemorrhagic Hydrocephalus (TROPHY) international prospective database seeks to compare various temporizing measures and is expected to yield additional multicenter information on NEL safety, efficacy, and neurodevelopmental outcomes [36], and has encouraging 6-month follow-up results so far [37].

Limitations include those inherent to a single-institution retrospective series with a small sample size and relatively short follow-up as our cohort matures. Neurodevelopmental outcomes at 2- and 5-year follow-up will be essential. In addition, our center included three neurosurgeons with evolving access to four endoscopes in the timeframe of the study [38]. D’Arcangues et al. only used rigid endoscopes with inflow/outflow channels, while the ongoing ENLIVEN-UK randomized controlled trial recruiting across 11 UK centers does not prescribe a standard endoscopic choice [12, 39]. For each center, experience using neuroendoscopy, development of standardized perioperative protocols, and multidisciplinary collaboration of neonatology, neurology, neurosurgery, and neuroanesthesiology teams are essential.

## Conclusions

This study shares our single US institution’s initial NEL experience for IVH of prematurity and PHH in a group with greater comorbidities and lower mean GA and birth weight compared with published European cohorts. Further studies are necessary to define the risks and benefits of NEL, the criteria for optimal case selection, and the most appropriate outcome measures, including long-term neurodevelopment.

## Supplementary Information

Below is the link to the electronic supplementary material.

Supplementary Figure 1 (PNG. 653 KB )

High Resolution Image  (TIFF. 8.22 MB KB) 

## Data Availability

All relevant data are included in the manuscript body.

## Abbreviations

NEL: Neuroendoscopic lavage
IVH: Intraventricular hemorrhage
GA: Gestational age
kg: Kilograms
BW: Birth weight
PHH: Posthemorrhagic hydrocephalus
VSGS: Ventriculosubgaleal shunts
VAD: Ventricular access devices
DRIFT: Drainage, irrigation, and fibrinolytic therapy
HCRN: Hydrocephalus Clinical Research Network
CSF: Cerebrospinal fluid
vEEG: Video electroencephalography
HUS: Head ultrasound
ETV: Endoscopic third ventriculostomy
HMD: Hyaline membrane disease
PDA: Persistent ductus arteriosus
NEC: Necrotizing enterocolitis
FOHR: Frontal occipital horn ratio
VI: Ventricular index
VPS: Ventriculoperitoneal shunt
NICU: Neonatal intensive care unit
SOPHH: Shunting outcomes in posthemorrhagic hydrocephalus
TROPHY: Treatment of post-hemorrhagic hydrocephalus
