# Supplementary figures and images for: Neuroendoscopic lavage: a single-center retrospective cohort in the USA
Source: Childs Nerv Syst. 2025 Nov 13;41(1):353. doi: 10.1007/s00381-025-06994-z (PMC12615566; doi:10.1007/s00381-025-06994-z)

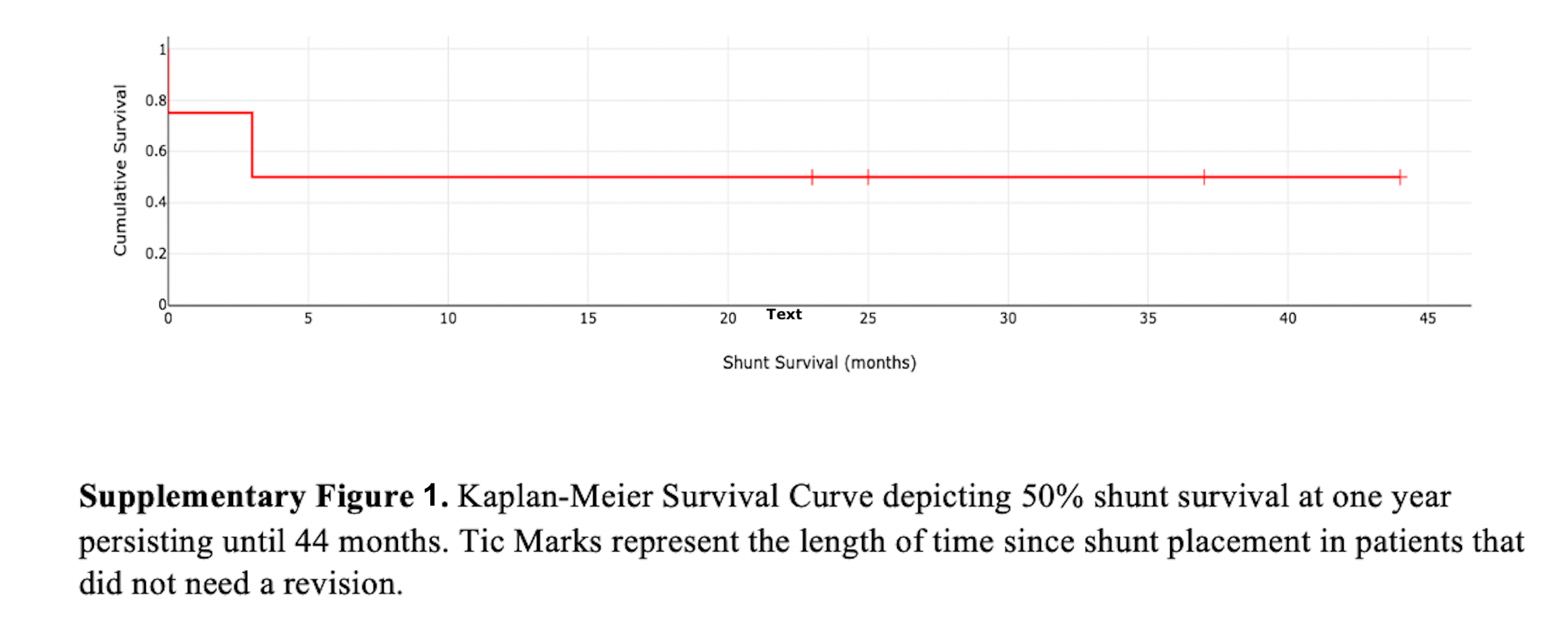

Supplement: Supplementary file 1 — Supplementary Figure 1 (PNG. 653 KB ) [file 381_2025_6994_Fig6_ESM.png]

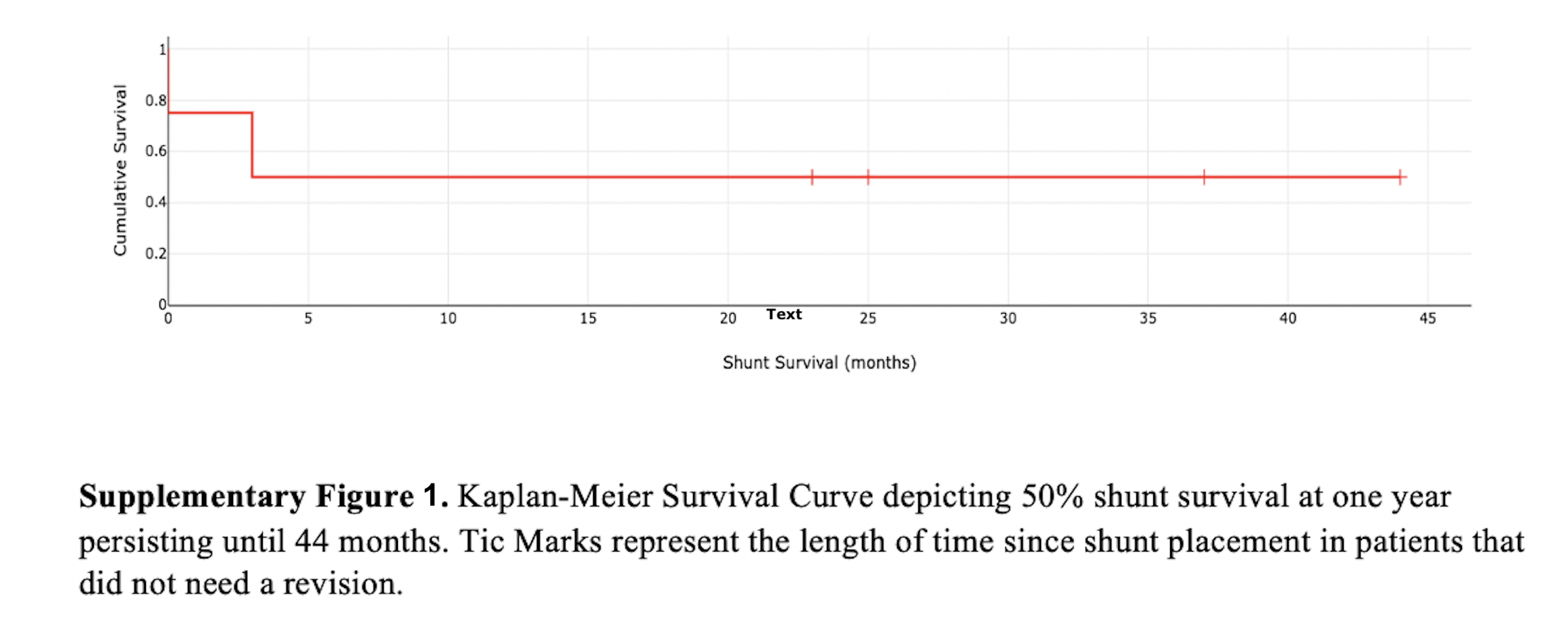

Supplement: Supplementary file 2 — High Resolution Image (TIFF. 8.22 MB KB) [file 381_2025_6994_MOESM1_ESM.tiff]
